# Tailored delivery of analgesic ziconotide across a blood brain barrier model using viral nanocontainers

**DOI:** 10.1038/srep12497

**Published:** 2015-08-03

**Authors:** Prachi Anand, Alison O’Neil, Emily Lin, Trevor Douglas, Mandë Holford

**Affiliations:** 1 Hunter College-CUNY, Belfer Research Building, 413 E, 69th Street, New York, NY-10021 (USA); 2 The American Museum of Natural History, Central Park West 79th Street, New York, NY-10024 (USA); 3 Indiana University, 800 E. Kirkwood Ave., Bloomington, IN-47405 (USA)

## Abstract

The blood brain barrier (BBB) is often an insurmountable obstacle for a large number of candidate drugs, including peptides, antibiotics, and chemotherapeutic agents. Devising an adroit delivery method to cross the BBB is essential to unlocking widespread application of peptide therapeutics. Presented here is an engineered nanocontainer for delivering peptidic drugs across the BBB encapsulating the analgesic marine snail peptide ziconotide (Prialt®). We developed a bi-functional viral nanocontainer based on the *Salmonella typhimurium* bacteriophage P22 capsid, genetically incorporating ziconotide in the interior cavity, and chemically attaching cell penetrating HIV-Tat peptide on the exterior of the capsid. Virus like particles (VLPs) of P22 containing ziconotide were successfully transported in several BBB models of rat and human brain microvascular endothelial cells (BMVEC) using a recyclable noncytotoxic endocytic pathway. This work demonstrates proof in principle for developing a possible alternative to intrathecal injection of ziconotide using a tunable VLP drug delivery nanocontainer to cross the BBB.

Passage of endogenous and exogenous substrates in and out of the central nervous system (CNS) is controlled by the blood-brain-barrier (BBB). The BBB is the primary interface between the bloodstream and the brain parenchyma and is often an insurmountable obstacle for a large number of pharmaceutical drugs, including peptides, antibiotics, and chemotherapeutic agents^1^,^2^. More than 98% of candidate drugs for CNS diseases have been hampered by the poor permeability of the BBB, presenting a major challenge for the pharmaceutical industry^3^. Devising a robust delivery method for crossing the BBB is essential to unlocking the widespread application of peptidic therapeutics. Recent advances in nanotechnology have demonstrated that viral capsids are effective and efficient nanocontainers for drug delivery^4^,^5^. Diverse cargos, including diagnostic imaging agents, fluorescent dyes, gold particles, luminescent quantum dots, anticancer agents, as well as antigenic peptides have been incorporated into the cavities of various viruses for a range of biomedical applications^6^.Viral capsids and other nanocontainers offer a promising Trojan horse strategy for tailored peptide therapeutic administration and are applied here to demonstrate the delivery of venomous marine snail analgesic peptide ziconotide (Prialt®) across the BBB models (Fig. 1).

Venomous snail neuropeptides, due to their chemical and biological diversity, coupled with high specificity, affinity, and molecular recognition, are fortuitous therapeutic resources for manipulating signaling in the nervous system^7^,^8^. In particular, Conoidean marine snails (cone snails, terebrids, and turrids) express disulfide-rich neuropeptides in their venom to subdue prey, requiring the peptides to be fast acting, efficient, and highly specific – all essential virtues of a successful drug candidate^9^,^10^,^11^. Ziconotide (ω-MVIIA), a 25 amino acid peptide expressed in the venom of cone snail *Conus magus,* is an analgesic therapy commercially approved in the US and Europe^12^. MVIIA alleviates neuropathic pain associated with a wide range of conditions, including diabetes, shingles, leprosy, multiple sclerosis, HIV/AIDS, stroke, cancer, and nerve damage due to trauma or surgery^13^. In general, treatment of neuropathic pain presents a significant clinical challenge, as current therapeutics, including morphine, gabapentin and antidepressants, possess significant drawbacks, such as being ineffective in many patients, declining in efficacy over time with the development of tolerance, or producing severe side-effects, such as addiction^14^,^15^,^16^. Due to the side effects associated with opioid drugs, non-opioids have been pursued to discover novel analgesic therapies. MVIIA is unique in that it is the first and only non-opioid marine snail drug approved for the treatment of neuropathic pain. As MVIIA is specific to N-type calcium channels, it does not target opioid receptors, and as a result does not have the same side effects. MVIIA heralded a new paradigm for identifying analgesic compounds, namely, inhibitors of N-type calcium channels. Several other peptides from venomous cone snails are currently being investigated as therapies for neurological pain^17^,^18^,^19^.

Despite the promise of cone snail neuropeptides for pain treatment, their potential as therapeutics is limited by their inefficient systemic delivery methods^20^,^21^. Recent attempts to improve the oral delivery of cone snail peptides (conopeptides), such as synthetic cyclization, have improved structural stability and reduced peptide proteolysis^22^,^23^. However, the rigid disulfide-rich structural conformation of conopeptides is imperative to their function, and as such, decreased bioactivity after cyclization was observed. Similarly, widespread application of MVIIA is limited, as it does not cross the BBB and has to be applied by intrathecal injection^13^,^24^. Thus there is an urgent need for a reliable delivery system to improve the uptake of CNS active venomous marine snail neuropeptides in their native form.

Here we present a bifunctional viral nanocontainer, constructed through chemical and genetic manipulation of the *Salmonella typhimurium* bacteriophage P22 capsid and the HIV-Tat peptide, as proof-of-principle for effective delivery of MVIIA across the BBB (Fig. 1). The P22 viral nanocontainer was chosen as a model drug delivery device as it efficiently self assembles, and the interior and exterior of the capsid are readily applicable to either chemical or genetic modifications to produce the desired designer peptide-nanocontainer conjugate. It has been previously demonstrated that the conjugation of the protein transduction domain of transcriptional activator human immunodeficiency virus type-1 HIV-Tat peptide (YGRKKRRQRRR) to proteins with molecular weights ranging from 36 to 119 kDa, or inorganic quantum dots, enhances uptake across the BBB via adsorptive mediated transcytosis^25^,^26^,^27^. At ~54 nm in diameter, P22 capsids are significantly larger than the proteins and quantum dots previously translocated and reported in the literature. However, our results indicate that Tat-P22-MVIIA conjugated virus like particles (VLPs) successfully translocated in *in vitro* and *in vivo* BBB mimics. This is the first demonstration of delivery of MVIIA across a modeled BBB using a nanoparticle delivery system and presents an alternative route to intrathecal injection. The results presented are promising towards the development of a tunable VLP nanocontainer for the delivery of peptide therapeutics across the BBB.

## Results and Discussion

### Construction of P22-MVIIA capsids

**Figure 2:**
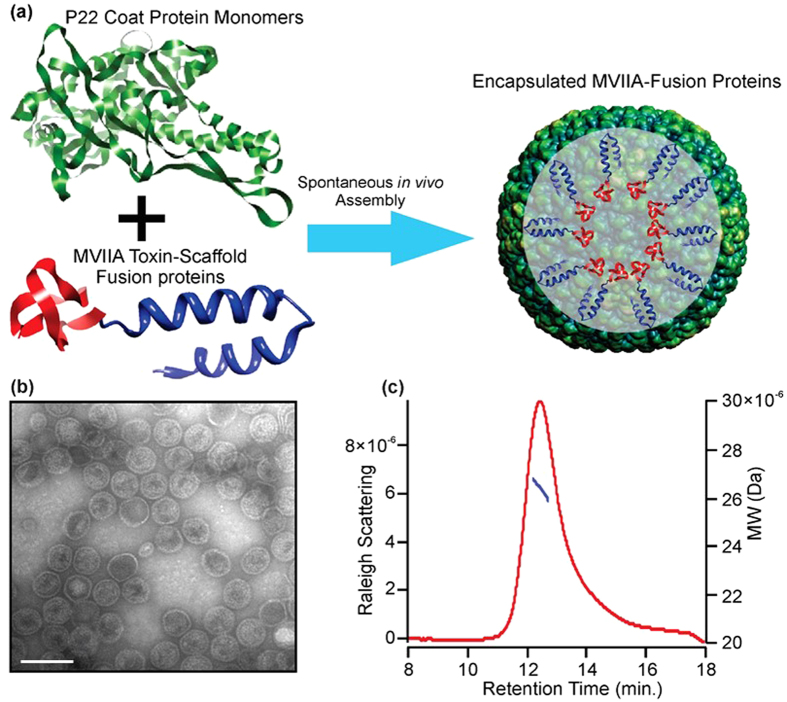
Construction of P22-MVIIA nanocontainers. (**a**) Illustration of the spontaneous *in vivo* assembly of the P22 coat protein monomers (green) with the MVIIA (red) scaffold (blue) protein fusion to form the fully encapsulated fusion proteins. (**b**) The assembled capsids are homogeneous in size, shape, and packaging by TEM (Scale bar: 100 nm) and (**c**) HPLC-MALS. HPLC-MALS shows the tight SEC elution profile (red) and the narrow range of total particle molecular weight (blue) across the peak.

The P22 capsid is an ideal drug delivery vehicle, as it has a constrained interior space amendable for assembly and specific packaging of bioactive cargo, a large exterior surface area that is conducive to multivalent genetic and chemical modifications for cell recognition and attachment, and, importantly, it can be made in large quantities^28^. Construction of modified P22-MVIIA capsids was performed using recursive PCR to add the MVIIA (CKGKGAKCSRLMYDCCTGSCRSGKC) sequence to the N-terminus of the P22 scaffold protein (Fig. 2a). A truncated version of the P22 scaffold protein, incorporating amino acids 238–303 (SP238), was utilized, as it has been shown that this truncated scaffold protein allows packaging of greater numbers of the fusion protein into the capsid. The P22-MVIIA construct was purified as previously described and expressed in the M338C P22 mutant coat protein background, which replaces a methionine (M) with a cysteine (C) residue at the exterior of the capsid, introducing a functional group for downstream chemical modifications^28^. Simultaneous induction of both the MVIIA-SP238 scaffold fusion protein and the M338C P22 coat protein in *E. coli* resulted in the spontaneous *in vivo* assembly of P22 capsids containing multiple copies of the MVIIA peptide. The P22-MVIIA capsids are morphologically indistinguishable from wild-type P22 capsids and are homogenous in size, shape and packaging as confirmed by transmission electron microscopy (TEM) and multiangle light scattering (MALS) (Fig. 2b,c). On average approximately 543 MVIIA-fusions (weighing ~10, 236.0 Da each) are encapsulated within each P22 capsid. This estimate was obtained by subtracting the theoretical molecular weight of the capsid (~19.6 MDa) from the total molecular weight of P22-MVIIA (25.2 MDa) as determined by MALS. The loading amount of MVIIA fusions are a significant number, ensuring an effective concentration of the expressed peptide.

To further confirm the presence of folded MVIIA-SP238 fusion protein, the capsids were heated to 75 °C, which induces a well-characterized morphological transition where the capsid expands and the 12 pentamers of the icosahedron dissociate, releasing the fusion protein contained inside^28^. LC-MS analysis of preheated P22-MVIIA capsids revealed a 10, 267.9 Da mass for the MVIIA-SP238 fusion protein, indicating that the MVIIA peptide was in a folded conformation. The observed mass of 10, 267.9 Da is consistent with a potassium adduct of the MVIIA-SP238 fusion protein, with the loss of six hydrogen atoms, corresponding to the formation of 3 disulfide bonds in the MVIIA peptide (Supplementary Fig. 1). While it is not possible to determine if the MVIIA expressed as a protein fusion is in its native form, previous attempts at recombinant expression and oxidative folding experiments of chemically synthesized cone snail neuropeptides have been shown to fold into native conformations^29^.

### Construction of Tat conjugated P22-MVIIA nanocontainers

**Figure 3:**
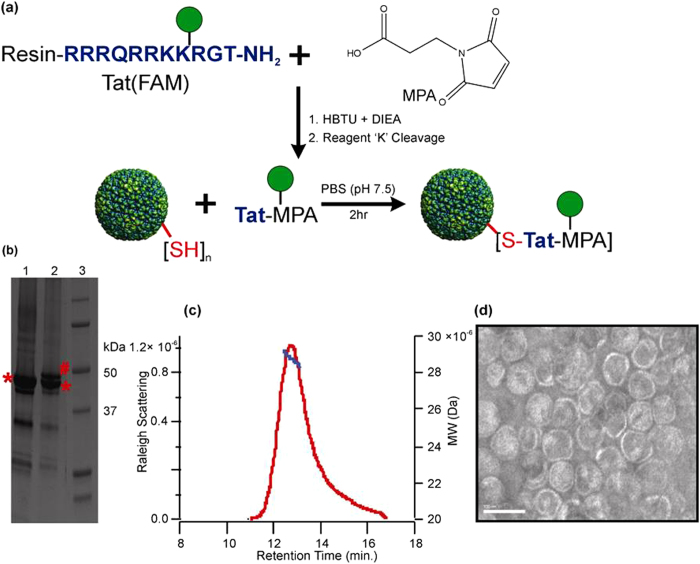
Conjugation of Tat (FAM) peptide to P22-MVIIA VLPs. (**a**) Schematic representation of the synthesis and conjugation procedure. Synthesized Tat peptide, bearing a fluorescent Fluorescein isothiocyanate (FAM) molecule (green circle), is activated with maleimidopropionic acid (MPA). The activated peptide is then conjugated via a free thiol group in M338C P22 mutant coat protein onto the VLP exterior. (**b**) PAGE showing the unmodified coat protein (*46.5 kDa) in lane 1, the increase in MW of the modified coat protein band (#48.7 kDa) in lane 2, and a molecular weight marker in lane 3. (**c**) HPLC-MALS data showing the average MW (blue) and the SEC elution profile (red) of the conjugated nanocontainer. (**d**) TEM image of conjugated Tat(FAM)P22-MVIIA nanocontainers indicating the integrity of the constructed VLPs. Scale bar: 100 nm.

Purified P22-MVIIA capsids were successfully conjugated via the M338C P22 coat protein exterior cysteine to a chemically synthesized fluorescein labeled HIV-Tat (Tat (FAM)) peptide (Fig. 3). Tat(Fam) was chemically synthesized using Fmoc solid phase peptide synthesis and purified by reverse-phase High Performance Liquid Chromatography (HPLC) and modified with maleimidopropionic acid (MPA) for conjugation to P22-MVIIA capsids in solution (Fig. 3a, Supplementary Fig. 2). Construction of the Tat(FAM)-P22 coat protein construct was confirmed by denaturing (SDS) gel electrophoresis (Fig. 3b), where a visible shift in the conjugated Tat(FAM)-P22 coat protein band was observed compared with the non-conjugated P22 coat protein construct. Densitometry of the shifted Tat(FAM)-P22 coat band compared with unmodified P22 coat band indicated that ~40% of the coat proteins were conjugated with the Tat peptide. Size exclusion chromatography (SEC-HPLC) coupled with MALS detection of the Tat(FAM)-P22-MVIIA capsids exhibited no change in capsid morphology (by the radius of gyration) and only slight variation in the average total particle molecular weight compared with the unmodified VLPs, within the error of the measurement (Fig. 3c). This is consistent with the expected small mass increase associated with attachment of the peptide to roughly 40% of the subunits. Direct visualization of the particles by TEM confirmed that P22 particle morphology was unaffected by the conjugation (Fig. 3d). The Tat(FAM)-P22-MVIIA conjugation reaction was further confirmed by LC-MS analysis. A mass of 48,705.2 Da was observed, which corresponds to the addition of the mass of the Tat(FAM)-MPA peptide (2,137 Da) to the M338C P22 coat protein (46,568.0 Da,) (Supplementary Fig. 3).

### Cellular uptake and translocation of conjugated VLPs in *in-vitro* BBB models

The cellular uptake of Tat(FAM)-P22-MVIIA conjugated VLPs was initially characterized using an *in vitro* Rat Brain Microvascular Endothelial Cells (RBMVEC) BBB model. RBMVECs are well-characterized cell systems and are routinely used to screen peptide drugs for their potential to cross the blood-brain barrier^30^,^31^. The integrity of the BBB mimic was confirmed by expression of tight junction proteins zona occludens 1 (ZO-1) and permeability glycoprotein (Pgp) in 60–70% confluent RBMVECs (Supplementary Fig. 4). ZO-1 is one of several scaffolding proteins considered to be important in signal transduction and in linking tight-junction-associated transmembrane proteins^32^,^33^. Pgp has a profound effect on the entry of drugs, peptides and other substances into the CNS^34^,^35^. Western blot analysis confirmed the presence of Pgp in our BBB mimic. Similarly, immunostaining of rat ZO-1 confirmed the formation of intercellular tight junctions in RBMVECs. Efficient uptake of Tat(FAM)-P22-MVIIA VLPs by RBMVEC monolayer, was observed by fluorescence microscopy (Supplementary Fig. 5). RBMVECs were incubated for 20 min with conjugated VLPs, after which bright green fluorescence was observed inside the cells using spinning disc microscopy. Transfer of the large P22 capsid (54 nm) across the endothelial cells of the BBB mimic is in agreement with several previous reports that suggest Tat is an effective tool for maneuvering the BBB^25^,^36^,^37^.

A dynamic *in vitro* BBB (DIV-BBB) model of human microvascular endothelial cells, in association with human astrocytes, was used to further characterize the translocation of P22-MVIIA VLPs^37^,^38^,^39^. In this model, the conjugated P22-MVIIA VLPs (referred to as bolus material) is administered to the luminal chamber (LC) and transclocation is measured by trans-endothelial electrical resistance (TEER) across the human BMVE cells to the extraluminal chamber, also referred to as the extracellular space (ECS) (Supplementary Fig. 6a).

10 μg/ml final concentration of P22-MVIIA VLPs bolus material was applied to the DIV-BBB model and samples were then collected over time from the LC and the ECS and analyzed by ESI-LC/MS. P22-MVIIA VLP specific m/z ions were initially present only on the luminal side of the DIV-BBB model, but after 15 min, these ions could be detected in the ECS, suggesting that the P22-MVIIA VLP had begun to pass through the BBB mimic (Supplementary Fig. 6b and Table 1). While TEER experiments generally use radiolabeled bolus material, our P22-MVIIA construct was not radiolabeled, therefore it was not possible to obtain a quantitative measure of the transport rate to the lower compartment. However, as an alternative, mass spectrometry was used to monitor the reaction and determine presence/absence of the P22-MVIIA VLPs bolus material in the LC and ECS compartments. To assess the functionality and integrity of the DIV-BBB used in the P22-MVIIA construct experiment, a bolus (0.5 ml) of radioactive tracer [^3^H]-sucrose was applied into the LC and diffusion into the ECS was monitored over time (Supplementary Fig. 6c). After 10 min [^3^H]-sucrose crossed the BBB endothelial cells in a manner similar to our P22-MVIIA virus like particles. Taken together, *in vitro* RBMVEC and DIV-BBB findings indicate P22-MVIIA VLPs are being transported across BBB models.

### Mechanism of the P22-MVIIA conjugated VLPs BBB transport

**Figure 4:**
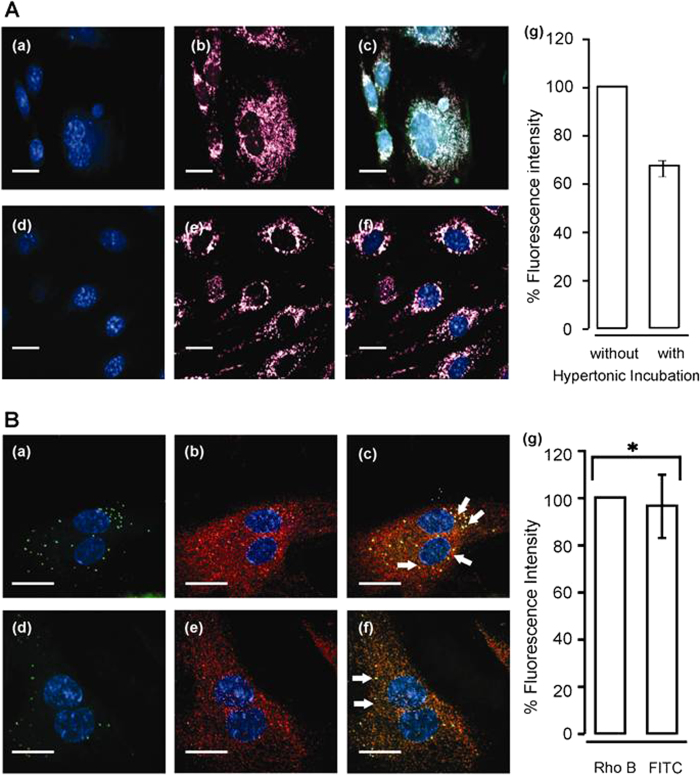
(A) Endocytic uptake of Tat (FAM)-P22-MVIIA VLPs by RBMVE cells. Spinning disc microscopy observations of RMBVE cells after 20 min incubation with conjugated VLPs using: (a,d) DAPI filter. (b,e) RhoB filter for lysotracker (Excitation/ Emission: 490/525 nm). (c,f) Overlay of DAPI, RhoB and FITC channels showing the co-localization of conjugated VLPs with acidic organelles. Cells with hypertonic solution (0.4 M sucrose) show reduced uptake of conjugated VLPs, but still the absolute presence of lysosomes (e–f). Scale bar = 12 μm. (g) The reduced cellular uptake of conjugated VLPs plotted by measuring the green fluorescence intensities for the conjugated VLPs of the cells incubated 1 without and 2 with hypertonic solution. Ten different regions were selected and fluorescence intensities were measured. 2 were corrected relative to the value obtained from the image 1 (n ~ 100). (**B**) Tat (FAM)-P22-MVIIA VLPs in recycling pathway as indicated by Rab11 Immunostaining. (a–f) Distribution of Rab11, which is known to associate primarily with perinuclear recycling endosomes and regulate recycling of endocytosed proteins. Immunoreactivity in fluorescent VLPs treated cells stained for Rab11 are indicated as follows: (a,d) = DAPI and FITC filter. (b,e) = RhoB and DAPI. And (c,f) = overlay images with all channels on (DAPI, RhoB and FITC). Arrows in images (c) and (f) show the concentrated VLPs in recycling endosomes due to co-localization with Rab11. (g) Quantitation of images for green (FITC) and red (RhoB) filters shown in (A–F). *p = 0.55 or p > 0.05. Scale bar: 12 μm.

There are several receptor-mediated endocytic pathways, such as clathrin-mediated endocytosis, caveolae-mediated endocytosis, macropinocytosis, and clathrin- and caveolae-independent endocytosis, that would lead to conjugated P22-MVIIA VLPs trafficking in the BBB^40^,^41^,^42^. Several *in vitro* experiments were performed to determine if the P22-MVIIA conjugated VLPs were translocating the RBMVEC monolayer via a receptor-mediated endocytosis pathway. During endocytosis molecules are internalized in vesicles and are directed to endosomes or lysosomes within the cell. To identify if the modified capsids were being transported via endocytic vesicles in endothelial cells, RBMVECs were incubated with P22-MVIIA VLPs together with a dye specific to acidic organelles such as endosomes and lysosomes (LysoTracker red dye, Life Technologies). Spinning disc confocal microscopy using RhoB and FITC filters to visualize RBMVECs incubated with lysotracker displayed a strong pink fluorescence staining of acidic vesicles in the cells (Fig. 4A). An overlay image of all channels confirmed the co-localization of fluorescence from conjugated VLPs and the lysotracker dye, suggesting that the modified P22-MVIIA capsids are being translocated via acidic lyosomal or late endosome vesicles. RBMVECs pre-incubated with hypertonic solution (0.4 M sucrose) that non-specifically blocks clathrin-mediated endocytosis (CME) pathways^43^ displayed approximately 35% reduced uptake of conjugated VLPs compared to that of untreated RBMVECs (Fig. 4A(g)). These results indicate that translocation of conjugated VLPs is mediated by more than one uptake mechanism and not solely dependent on the clathrin-mediated pathway. It has been previously demonstrated that P22 capsids are resistant to degradation by proteases and are relatively stable down to pH 4. When the conjugated VLPs were exposed to buffer solution of pH 4, they remained stable for up to 24 h as confirmed by LC-MS and DLS analyses (data not shown). It is thus expected that translocation via acidic organelles, such as late endosomes or lysosomes (pH ~ 4.8), should not affect the integrity of the P22-MVIIA nanocontainers *in vivo*.

Transport of P22-MVIIA VLPs via endocytic vesicles can lead to lysosomal degradation, accumulation in late endosome, or recycling out of the cell via recycling endosomes. To determine if the conjugated P22-MVIIA VLPs were accumulating in late endosomes or recycled out of the RBMVECs BBB mimic, imunofluorescence assays were performed with Rab 11 protein, which is known to associate primarily with perinuclear recycling endosomes and to regulate recycling of endocytosed proteins^44^. The majority of FITC fluorescence associated with P22-MVIIA VLPs co-localized with that of Rab11 (Fig. 4B).

Quantitation of the co-localized green fluorescence indicated a high correlation coefficient (~0.84 ± 0.06), implying significant co-localization (Fig. 4B(g)). Co-localization of P22-MVIIA VLPs and Rab 11 protein strongly indicate that the conjugated capsids are not accumulating in lysosomes, but rather are being trafficked across the endothelial layer forming BBB via an endocytic pathway.

### Exposure to conjugated VLPs does not lead to cytotoxicity of RBMVECs

P22-MVIIA conjugated VLPs were not cytotoxic to the RBMVEC BBB model. *In vitro* cytotoxicity analysis in RBMVECs was performed using a colorimetric assay of tetrazolium dye MTT 3-(4,5-dimethylthiazol-2-yl)-2,5-diphenyltetrazolium bromide^45^. RBMVECs were incubated with increasing concentrations of Tat(FAM)-P22-MVIIA capsids (6.25 μg/ml, 12.5 μg/ml, and 20 μg/ml) for 24 h. No significant viability difference was observed between the conjugated treated RBMVECs and untreated RBMVECs in the MTT assay (Supplementary Fig. 7). Incubation with conjugated VLPs did not cause any severe toxicity up to 20 μg/ml, which is 2x the 10 μg/ml concentration used in all experiments. These findings indicate that the P22-MVIIA VLPs are biocompatible, which is in agreement with previous studies demonstrating the suitability of P22 capsids for *in vitro* and *in vivo* applications^46^.

### *In vivo* application of P22-MVIIA conjugated VLPs

Successful *in vivo* application of P22-MVIIA VLPs would require that the engineered VLPs are neutral to blood complement and can be readily passaged through systemic circulation. To provide proof of biocompatibility and to determine systemic pharmacokinetics of Tat-P22-MVIIA conjugated nanocarriers, nude mice expressing malignant subcutaneous tumors were injected intravenously and characterized using *ex vivo* organ epifluorescence at various time points (Supplementary Fig. 8). Two to 5 h after initial injection into the tail vein of the mice, conjugated Tat-P22 MVIIA nanocarriers were imaged in excised tumors. A positive control of the Tat peptide labeled with fluorescein (Tat-FAM) was also injected and similarly imaged. Both Tat-P22 MVIIA and Tat-FAM constructs displayed a high degree of epi-fluorescence compared to the injections of PBS solution. While this experiment does not demonstrate uptake into the CNS it does confirm that conjugated P22-MVIIA nanocarriers are being successfully trafficked in cells and will not be degraded in the blood. Additional experiments are necessary to determine *in vivo* CNS activity and the therapeutic activity of the encapsulated MVIIA peptide.

In summary, a bi-functional drug delivery nanocontainer was designed using genetic and chemical manipulation of *Salmonella typhimurium* bacteriophage P22 capsids to incorporate the analgesic neuropeptide ziconotide (MVIIA) within the interior cavity, and the cell penetrating HIV-Tat peptide on the exterior of the capsid. Tat modified P22-MVIIA capsids (conjugated VLPs) were successfully taken up in an *in vitro* RBMVEC-BBB model via an endocytic pathway, which was further confirmed by the use of DIV-BBB with human microvascular endothelial cells, and in an *in vivo* mice application to determine biocompatibility. Characterization of the analgesic drug delivery nanocontainers by multi-angle light scattering (MALS), liquid chromatography mass spectrometry (LC-MS), and transmission electron microscopy (TEM) data indicated the P22-MVIIA capsids were highly stable and homogeneous. Importantly, MVIIA expressed in the interior of P22 cavity was disulfide folded as confirmed by LC-MS. Taken together, these findings provide proof-of-principle for the use of viral nanocarriers to deliver peptide therapeutics in the central nervous system.

By leveraging the specificity, diversity, and potent biological activity of peptide natural products, such as venomous marine snail neuropeptides, with VLPs, it was possible to develop a peptide-drug delivery system applicable for investigating neuropathic pain disorders. Although the marine snail neuropeptide MVIIA was used in this demonstration, the VLP peptide delivery system described would be universally applicable to the delivery of other peptidic natural product therapeutics.

## Methods

### Molecular construction of self-assembled nanocontainer (P22-MVIIA-SP238)

#### Molecular Construction

Using a recursive PCR strategy, the gene for MVIIA was added on to the N-terminal end of the scaffold protein fusion^28^. A truncated form of the P22 scaffold protein was used that encodes for amino acids 238–303 (SP238). In this strategy, primers are designed such that the MVIIA gene is added in two PCR amplification steps. Detailed purification methodology is provided in the supplementary information.

### Construction of conjugated VLPs

#### Synthesis of fluorescent Tat (Tat-FAM) peptide

Tat peptide with a sequence of YGRKKRRQRRR was synthesized by Fmoc solid phase peptide synthesis (SPPS) on a CEM Liberty synthesizer using standard side chain protection. At position 8 in place of Fmoc-Lys (Boc)-OH, Fmoc-Lys (5/6 FAM)-OH was used to attach a fluorescein tag to the Tat peptide. Following the synthesis of Tat-fam peptide at the N-terminal of the chain 3-Maleimidopropionic acid was attached by a condensation reaction and then by a treatment with Reagent K [92.5% TFA (trifluoroacetic acid), 2.5% TIS (Triisopropylsilane), 2.5% EDT (Ethanedithiol) and 2.5% water, 3 h)] and cold ether precipitation, Tat-FAM was purified using a method of 20% buffer B (80% acetonitrile in 20% water, 0.1% TFA) to 30% buffer B in buffer A (0.1% TFA in water) in 30 min on RP-HPLC (Agilent 1260) and mass characterized by ESI-LCMS (Agilent Mass Q-TOF LC/MS 6520).

#### Tat-FAM conjugation to the VLPs

The construct of viral nanocapsid was made in M338C coat protein background. This coat protein exposed a cysteine to the outside for chemical modification. 1:50 molar ratio of coat protein subunit of nanocapsid to maleimide functionalized Tat(FAM) peptide were incubated at 37 °C for 2 h on a shaker for conjugation. Conjugation reaction was confirmed by SDS-PAGE analysis followed by ESI-LCMS, HPLC-MALS and TEM.

### Transmission electron microscopy (TEM) of conjugated VLPs

Samples (2 μl, 0.1 mg/ml) were applied to Formvar coated Nickel grids and incubated for 30 sec, excess liquid was removed with filter paper, this step was repeated twice. Grids were then stained with 2 μl of 1% Uranyl acetate, and excess stain was removed with filter paper. Images were taken on a JEOL JEM-100CX II TEM at accelerating voltage of 100 kV.

### SDS-PAGE (polyacrylamide gel electrophoresis) of conjugated VLPs

Samples were separated on a 10% denaturing gel using a constant voltage of 150 volts. Gels were stained with coomassie brilliant blue stain, rinsed with water, and destained. Images were recorded using a Fotodyne machine.

### Delivery of conjugated- VLPs into RBMVEC

#### Cell Culture

Rat brain microvascular endothelial cells (RBMVEC) and required cell culture reagents were acquired from Cell Applications, Inc. Prior to the seeding of cells, the cell culture flasks were coated with an attachment factor solution overnight at 37 °C. Cells were grown at 37 °C in 5% CO_2_ in Rat Brain Endothelial Cell Growth medium (RBECGM). RBMVEC were not grown nor used past the 7^th^ passage.

#### Cellular uptake of conjugated VLPs into the RBMVEC using Spinning disc confocal microscopy

RBMVEC were seeded at a cell density of 0.3 × 10^6^ in 35 mm dishes. Tat conjugated VLPs at a final concentration of 10 μg/ml was added to each dish and incubated for 20 min at 37 °C in 5% CO_2_. After 3 washes with PBS for 10 min each, cells were fixed using 4% paraformaldehyde (PFA) for 20 min. Coverslips were mounted onto glass slides with ProLong Antifade Reagent with DAPI (Life Technologies). Images were taken using the Perkin Elmer Ultra VIEW ERS spinning disc confocal microscope. Five to ten fields chosen at random were imaged for each sample and representative images are shown for each experiment. DAPI was used to stain for the presence of the nuclei as a counter stain. A Fluorescein isothiocyanate (FITC) filter was employed to image RBMVEC incubated with conjugated VLPs.

To inhibit endocytosis, prior to the incubation with VLPs, cells were incubated with hypertonic sucrose solution (0.45 M) for 15 min at 4 °C and then returned for 120 min at 37 °C and then VLP treatment is given as mentioned above and slides were prepared and imaged.

#### Immunofluorescence assay for Rab11, a marker of recycling endosomes

RBMVECs were grown on 10 mm cover slips in 4 well plates. All steps were performed at RT (room temperature) unless otherwise mentioned. After the 20 min treatment with conjugated VLPs the cells were washed with PBS, fixed and then permeabilized with 0.5% tritonX-100 in PBS at RT for 10 min. Following incubation with a blocking solution containing 5% goat serum and 0.3% tritonX-100 in PBS for 20 min, cells were incubated with primary antibodies Rab11 (Mouse Monoclonal Antibody- Life technologies) at 4 °C overnight, washed with PBS, and probed with the required RhoB-conjugated secondary antibody (Goat Anti-Mouse IgG (H + L) Antibody, Life Technologies). The treated coverslips were mounted onto a slide with Prolonged Gold antifade reagent with DAPI (Life Technologies). Fluorescence images were taken by using a Perkin Elmer UltraView ERS a spinning disk confocal microscope.

### DIV-BBB (dynamic *in vitro*-blood brain barrier) cellular uptake and permeability characterization by LC-MS

Cellular uptake assay was performed at Cleveland Clinic, Ohio and the samples were characterized by LC-MS in the Holford laboratory (Supplementary Table 1). Normal HBMVEC along with human astrocytes were used to form the BBB. Transendothelial electrical resistance (TEER) was measured by real-time computerized monitoring systems between luminal and extraluminal chambers. Two separate cartridges were used to confirm the integrity and to perform the permeability assay in duplicate. Over the first 18 days of DIV-BBB setup, the TEER (trans endothelial electrical resistance) significantly changed from 1300 Ω cm^2^ to 1900–2000 Ω cm^2^, a change of 650 Ω cm^2^. After 21 days, functional assessment was performed by sucrose permeability and the average value calculated was 8.83 ± 0.89 × 10^−5^ cm min^−1^ for two different cartridges showing the lower permeability for sucrose, hence demonstrating the tight barrier formation in DIV-BBB. After the confirmation of integrity of BBB, conjugated VLPs were applied as bolus and samples were taken from extracellular (ECS), also referred to extraluminal, space and lumen (100 μl) at time zero, 10, 15, 30 and 60 min after the injection. Samples from ECS and lumen were subjected to LC/MS analysis to look for Tat(FAM)-P22-MVIIA conjugated coat protein subunit mass of conjugated VLPs (Supplementary Table 1 and Supplementary Fig. 6).

### Cellular toxicity of VLPs by MTT assay

The cytotoxicity of VLPs used was examined in RBMVEC by MTT assay. After 24 h incubation with conjugated VLPs, 20 μl of MTT solution (5.0 mg/ml) was added to each well and plates were incubated for 3 h at 37 °C in 5% CO2. After 3 h of incubation, medium containing MTT was aspirated and 100 μl of acidified isopropanol was added to each well and assayed in a PowerWave HT Microplate Spectrophotometer at 550 nm and 620 nm. The mean and standard deviation of the Delta OD values were calculated which were obtained by subtracting 620 nm values from 550 nm values. The absorbance of control cells was taken as 100% viability and the values of VLP treated cells were calculated as a percentage of control.

### Integrity of BBB in RBMVECs

#### Detection of Pgp expression by Western blot analysis

To confirm if tight junctions were formed in the cells used to visualize the trafficking of VLPs, the expression of Pgp in the cultured RBMECs was investigated by immunoblot analysis. Briefly, the cultured RBMVEC at different passages were harvested, and centrifuged to get a pellet. Washed pallets were homogenized in ice- cold cell lysis buffer containing 10 mM Tris– HCl, 1 mM MgCl_2_·6H_2_O, 1 mM EGTA, 1 mM mercaptoethanol, 1% glycerin, and protease inhibitor cocktail (1 mM dithiothreitol, 2 mM phenylmethylsulfonylfluoride). Cell homogenates were centrifuged at 11,000 rpm for 10 min at 4 °C. The supernatant was obtained as membrane fraction, and the protein concentration in this solution was measured with a Nanodrop (Thermo Fisher Scientific). An aliquot of cell sample was diluted with a volume of 2 × SDS sample buffer. The protein (10 μg) was separated by electrophoresis on 6% SDS-polyacrylamide gel and electrophoretically transferred to a PVDF (Millipore, MI, USA). The membrane was blocked by 5% dried non-fat skimmed milk in PBS containing 0.1% Tween-20 (PBST) at RT for 1 h and rinsed three times for 15 min in PBST. Afterwards, the membrane was incubated with the primary monoclonal antibody C219 (diluted 500-fold in PBST) overnight at 4 °C. After being rinsed with PBST, the membrane was incubated with the secondary polyclonal antibody (HRP-conjugated affinity-purified anti-mouse IgG (diluted 10,000-fold) in PBST at RT for 1 h and the signals were visualized by chemiluminescence. All blots were stripped and re-probed with polyclonal anti-β-actin antibody to ascertain equal loading of protein.

#### Immunofluorescence of ZO1 tight junction protein

Cells were grown on 10 mm cover slips in 4-well dishes and all steps were performed as earlier mentioned, except for the first conjugated VLPs incubation. The cells were incubated with primary antibodies (ZO-1 Mouse Monoclonal Antibody- Life technologies) at 4 °C overnight, washed with PBS, and probed with the required FITC-conjugated secondary antibody (Alexa Fluor® 488 Goat Anti-Mouse IgG (H + L) Antibody, Life Technologies). The treated coverslips of mono-layered cells were mounted onto a slide with Prolonged Gold antifade reagent with DAPI (Life Technologies). Fluorescence images were taken by using a Perkin Elmer UltraView ERS a spinning disk confocal microscope.

## Additional Information

**How to cite this article**: Anand, P. *et al*. Tailored delivery of analgesic ziconotide across a blood brain barrier model using viral nanocontainers. *Sci. Rep*. **5**, 12497; doi: 10.1038/srep12497 (2015).

## Supplementary Material

Supplementary Information

## Figures and Tables

**Figure 1:**
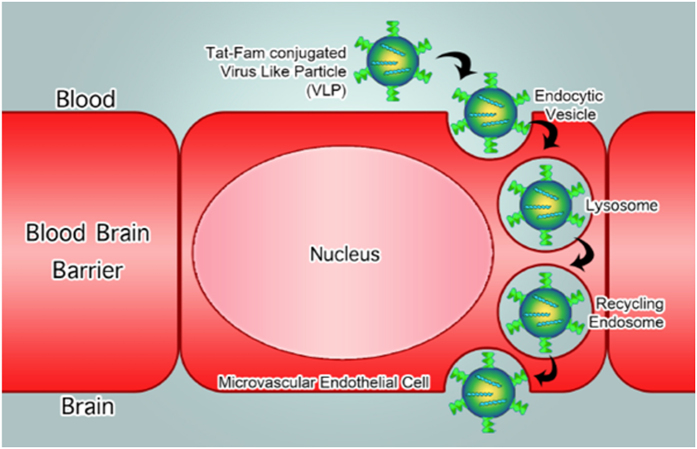
Trojan horse virus like particle design for delivering peptides across the BBB. Concept figure of engineered viral nanocontainers encapsulating marine snail peptide MVIIA in the interior and cell penetrating peptide Tat(FAM) on the exterior shuttle the nanocontainers across the BBB using an endocytic pathway.
