# Supplementary material for: Tailored delivery of analgesic ziconotide across a blood brain barrier model using viral nanocontainers
Source: Sci Rep. 2015 Aug 3;5:12497. doi: 10.1038/srep12497 (PMC4522602; doi:10.1038/srep12497)
Supplement: Supplementary Information [file srep12497-s1.doc]

**Supplementary Document**

**Tailored delivery of analgesic ziconotide across a blood brain barrier model using viral nanocontainers**

Prachi Anand^1, 2^, Alison O’Neil^3^, Emily Lin^1^, Trevor Douglas^3^, Mandë Holford^1, 2*^

^1^Hunter College-CUNY, Belfer Research Building, 413 E, 69^th^ Street, New York, NY-10021 (USA)

^2^The American Museum of Natural History, Central Park West 79th Street

New York, NY-10024 (USA)

^3^Department of Chemistry, Indiana University, 800 E. Kirkwood Ave.,

Bloomington, IN-47405 (USA)

***Corresponding author:**

Dr. Mandë Holford, PhD (Associate Professor)

Hunter College-CUNY, Belfer Research Building, 413 E, 69^th^ Street, New York, NY-10021 (USA)

Email: mholford@hunter.cuny.edu

Ph: +1 212-396-7024

Fax: +1 212-772-5332

**Supplementary Methods**

**Molecular construction of self-assembled nanocontainer (P22-MVIIA-SP238)**

*Molecular Construction*

Using a recursive PCR strategy, the gene for MVIIA was added on to the N-terminal end of the scaffold protein fusion. ^26^ A truncated form of the P22 scaffold protein was used that encodes for amino acids 238-303 (SP238). In this strategy, primers are designed such that the MVIIA gene is added in two PCR amplification steps. First, the C-terminal end of the MVIIA gene is appended to the N-terminal end of the SP238 gene using a forward primer containing a section of the MVIIA gene and a short section of the SP238 gene. The reverse primer anneals to the 3’ end of the SP238 gene and adds a new BamHI restriction enzyme site. The product of this amplification is then used for the second amplification step to build the MVIIA gene. The second forward primer contains the N-terminal half of the MVIIA gene, adds a 5’ XmaI restriction enzyme site and an overhang that anneals to the template (the product of first PCR) to join the MVIIA gene together. In this second reaction, the reverse primer stays the same. The product of this second PCR amplification yields the MVIIA-SP238 gene with a 5’ XmaI site and a 3’ BamHI site. The primers used for this are as follows with restriction enzyme sites in bold, the overlapping “joining” section underlined, and the MVIIA start codon in capitals. On the reverse primer, the annealing site for the scaffold protein is in italics.

Fwd Primer 1 (MVIIA-SP238-Fwd Primer): tatgattgctgtaccggtagctgccgcagcggcaagtgc**ccatgg**tgtactcgactatccgaa

Fwd Primer 2: aatcat**cccggg**ATGtgcaaaggcaaaggtgcgaagtgcagccgcctgatgtatgattgctgtacc

Reverse Primer: aaaa**gagctc***ttatcggattccttt*

The resulting gene was digested with XmaI, SacI, and dpnI at 37°C for 3 h and cleaned up using a Qiagen reaction cleanup kit. The digested insert was ligated into the similarly digested and gel purified pET11a vector. The ligation product was transformed into XL2-Blue ultracompetent cells (Agilent, CA) and the subsequent colonies screened for insert via DNA sequencing. Positive clones were transformed into BL21(DE3) cells for expression.

*Protein Purification*

Transformed BL21 (DE3) *E. coli* were grown to an OD_600nm_ = 0.6 at 37°C. The culture was then induced with isopropyl β-D-1-thiogalactopyranoside (IPTG) (1 mM). The culture was allowed to grow an additional 4 h at 37°C with vigorous shaking. The bacteria were separated from the media by centrifugation at 4500 x rpm for 10 min. The cell pellets were resuspended in 50 mM phosphate, 100 mM NaCl pH 7.6 and frozen at -20°C overnight. The next day, the cell pellets were thawed and incubated with DNAse (20 mg/ml), RNAse (30 mg/ml), and lysozyme (15 mg/ml) (Sigma-Aldrich) for 30 min at room temperature. Cells were lysed further by sonication (40% duty cycle, 5 min three times with a 2 minute cool down between) on ice. The cell debris was removed via centrifugation at 14500 rpm for 25 min. The clarified lysate (25 ml) was then layered on top of a 35% (w/v) sucrose cushion (5 ml) and centrifuged at 48,000 rpm for 50 min in an ultra-centrifuge (Sorvall WX Ultra). The resulting virus pellet was resuspended in 50 mM phosphate 25 mM NaCl pH 7.2 and spun at 17,000 g for 20 min to remove any particulates and lipid. The virus was then dialyzed against the aforementioned buffer overnight.

*Size Exclusion Chromatography (SEC)*

Protein samples were additionally purified via SEC. Samples were centrifuged at 17,000 rpm for 10 min and the supernatant was loaded on to a preparative S-500 Sephadex (GE Healthcare) 80 ml column using an AKTA Pharmacia FPLC. The flow rate was 1 ml/min using 50 mM phosphate 25 mM NaCl (pH 7.2) and the total elution volume was 150 ml. Fractions were taken from the later half of the 60ml elution peak.

*Size exclusion chromatography - Multi angle light scattering (MALS)*

P22 capsid samples were separated by HPLC (Agilent 1200) size exclusion chromatography (WTC-0200S column Wyatt Technologies) at a flow rate of 0.7 ml/min. The mobile phase was phosphate buffer (50 mM, 100 mM NaCl, 200 ppm sodium azide, pH 7.2). Samples (25 μl) were injected on the column and run for 30 minutes and elution was monitored using UV-vis detector (Agilent), a refractive index detector (Wyatt), light scattering, and quasi-elastic light scattering was detected with a Dawn 8 (Wyatt). Average Mw, Rg, and R_H_ were determined from a fit of the data using a Zimmol plot in the Astra software from Wyatt.

**In vivo P22-MVIIA conjugated VLPs fluorescence imaging**

In order to determine the biocompatibility of Tat-P22-MVIIA conjugated nanocarriers, nude mice were injected intravenously with the imaging agent, sacrificed at various time points, and the *ex vivo* organ epifluorescence was quantified. MIA-PaCa2 cells were implanted subcutaneously (5 × 10^6^ cells in 200 μl 1:1 PBS/Matrigel (BD Biosciences, San Jose, CA)) in the shoulders of 7 mice and allowed to grow for approximately two weeks. Thereafter, for each time point 40mg/kg conjugated Tat-P22-MVIIA (in 150 μl of PBS), ~ 1.0 mg per mice of 20gm, was injected intravenously via lateral tail vein. As negative control, PBS (150 μl) was injected, and as a positive control 40 mg/kg Tat-FAM peptide (in 150 μl of PBS) was injected. The cohorts of mice were then sacrificed at select time points from 2 h or 6 h post injection. Tumor tissues were harvested post mortem and placed on a petri dish. Imaging and analysis for the epifluorescence imaging study was performed with the IVIS spectrum fluorescence imaging system (PerkinElmer) and Living Image 4.4 software.

**Supplementary figures - 9**

**Supplementary table-1**

**Figure 1. LC-MS analysis of MVIIA-SP238.** a) Amino acid sequence of the fusion protein (MVIIA is in bold face with the 6 cysteine residues in green). b) Mass spectrum of the fusion protein showing the observed mass as a potassium ion adduct of M^+11^  with the reduction of 6 hydrogen atoms. The 6H^+^ reduction is caused by the disulfide formation between the 6 cys residues of MVIIA . c) List of different isotopic ions of M^+11^.

**Figure 2. Characterization of Tat (FAM) peptide.** a) UHPLC of Tat-FAM peptide showing a single peak at 2.279min at 214 nm and b) at 450nm due to fluorescently labeled Lys at 8^th^ position in the sequence and c) Mass spectral characterization of Tat-FAM.

**Figure 3. LC-MS characterization of Tat (FAM) P22-MVIIA VLPs.** a). Mass spectral data for the conjugated coat protein subunit showing the different m/z for different ions, b). tabulated ions with all the charged species found during MS analysis with their abundances. c) Dynamic Light Scattering (DLS) results of non-conjugated nanocontainer with a diameter of 49.8 nm d) DLS of Tat (Fam)MPA conjugated nanocontainer with a diameter of 58.6 nm.

**Figure 4. Integrity of RBMVEC as *in vitro* BBB model.** Protein expression of rat ZO1 protein in RBMVEC. a-c) Immunofluorescence assay of passage 7 of ZO1 in RBMVEC. The flourescence is as follows: (a) DAPI (b) FITC and (c) overlay of DAPI and FITC images, Scale bar= 12μm. (d) Protein expression of rat P-glycoprotein in RBMVEC as determined by Western blot analysis, at passages 3 and 7 of cells. β-Actin was used as the control for loading quantity of the protein samples. (CF-cytosolic fraction, MF-membrane fraction) of different passage numbers (MF7/3=membrane fraction at 7^th^/3^rd^ passage, CF7=cytosolic fraction at 7^th^ passage).

**Figure 5.** **Cellular uptake of Tat(FAM)-P22-MVIIA.** a-c) *In vitro* spinning disc microscopy observations of RBMVECs cells after 20 min incubation with conjugated VLPs show bright green fluorescence inside the cells illustrating nanocontainer uptake. (a) DAPI filter. (b) FITC filter. (c) Overlay of DAPI and FITC image of cells incubated with conjugated VLPs. Scale bar=12μm.

**Figure 6. Passage of P22-MVIIA VLPs across BBB using dynamic in vitro blood brain barrier (DIV-BBB) model.** (a) Schematic of TEER experiment. (*) Denotes the luminal chamber (LC) and (+) denoted the extraluminal chamber (ELC), also referred to as the extracellular space (ECS). Human microvascular endothelial cells, in association with human astrocytes are expressed to in between the LC and ELC to model the BBB. (b) Permeability investigation in DIV-BBB by mass spectrometry. Examples of mass spectrum samples taken from Luminal (a) and ECS (b) at 60 min. (c) Measurement of functional integrity of DIV-BBB using ^3^H labeled sucrose. After 10 min the concentration of ^3^H labeled sucrose is shifted from luminal chamber (blue) to extracellular space (ECS) (red).

**Figure 7. Endocytic uptake of Tat (FAM)-P22-MVIIA by RBMVE cells**. Spinning disc microscopy observations of RBMCVE cells after 20 min incubation with conjugated VLPs using: (a,d) DAPI filter. (b,e) RhoB filter for lysotracker (Excitation/ Emission: 490/525 nm). (c, f) Overlay of DAPI, RhoB and FITC channels showing the co-localization of conjugated VLPs with acidic organelles. Cells with hypertonic solution (0.4 M sucrose) show reduced uptake of conjugated VLPs, but still the absolute presence of lysosomes (e-f). Scale bar=12 μm. g) The reduced cellular uptake of conjugated VLPs plotted by measuring the green fluorescence intensities for the conjugated VLPs of the cells incubated 1 without and 2 with hypertonic solution. Ten different regions were selected and fluorescence intensities were measured. 2 were corrected relative to the value obtained from the image 1. (n~100).

**Figure 8. Assessment of cytotoxicity of Tat(FAM)-P22-MVIIA VLPs in RBMVE cells.** In vitro cytotoxicity of conjugated Tat-P22-MVIIA VLPs in RBMVE cells using MTT assay. RBMVECs were incubated with increasing concentrations of Tat-P22-MVIIA VLPs for 24 h in a medium with serum. Control RBMVECs were not incubated with Tat-P22-MVIIA nanocontainers. No Significant difference from control was observed indicating the VLPs were not toxic to the *in vitro* BBB model used in this study.

**Figure 9. In vivo application of Tat(FAM)-P22-MVIIA conjugated nanocontainers.** Nude mice expressing MIA-PaCa2 subcuataneous tumor cells were injected intravenously in the tail with Tat(FAM)-P22-MVIIA constructs to determine biocompatibility of the constructed VLPs. Tumors were excised at various time points and ex vivo organ epifluorescence was quantified. In all images tumors are as follows: Top row, from left to right = PBS negative control at 2 h and Tat(FAM) peptide (positive control) at 2hrs. Bottom row from left to right = Tat(FAM)-P22-MVIIA nanocarriers at 2 h, 3 h, 4 h, and 5 h time points. a) Photo of the excised tumors. b) epifluorescence of excised tumors. c) Overlay of tumor photo and epifluorescence. Significant uptake of the Tat(FAM)-P22-MVIIA nanocarriers is witnessed in excised tumors compared to the PBS control which has negligible fluorescence**,** indicating systemic passage of the P22-MVIIAVLPs *in vivo.*

**Table 1.** Mass spectrometry analyses of Tat-P22-MVIIA VLPs in the DIV-BBB model.

**Figure 1.**

**a.** M**CKGKGAKCSRLMYDCCTGSCRSGKC**PWCTRLSERLTLKPRGKQISSAPHADQPITGDVSAANKDAIRKQMDAAASKGDVETYRKLKAKLKGIR

**b.**

Exp. mass=10235.0963Da

Obs. mass=10267.942Da [^+^K- 6(H^+^)]

**c.**

**
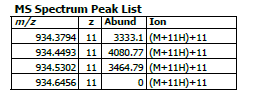
**

**Figure 2.**

**
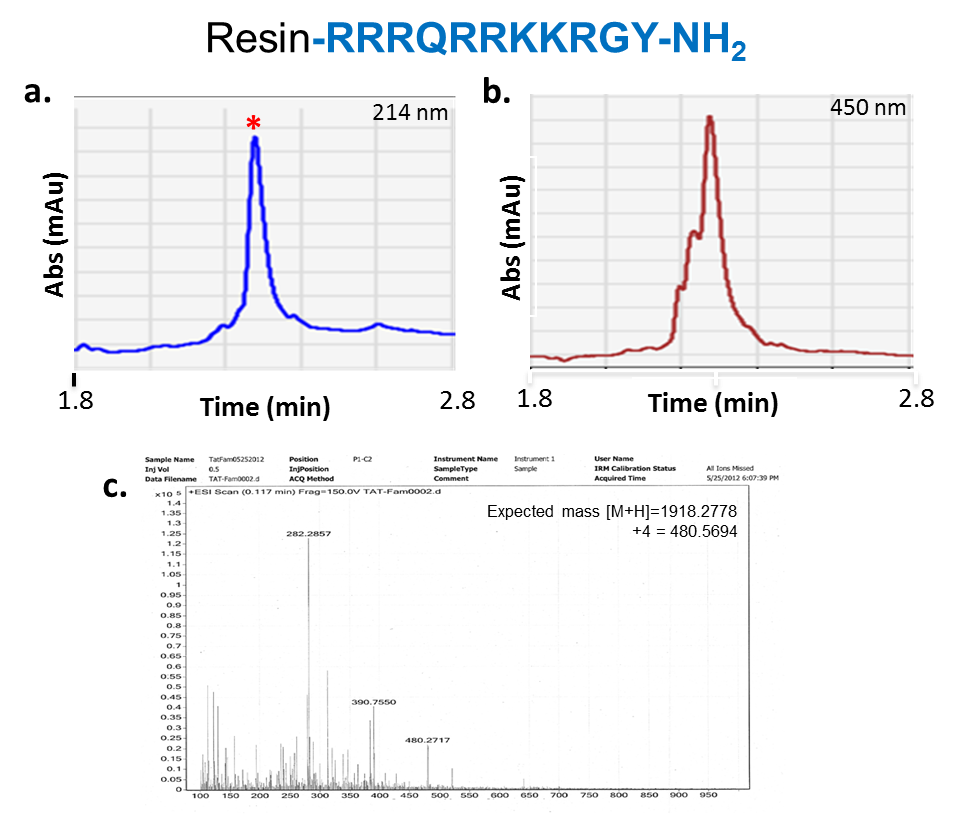
**

**Figure 3.**

**
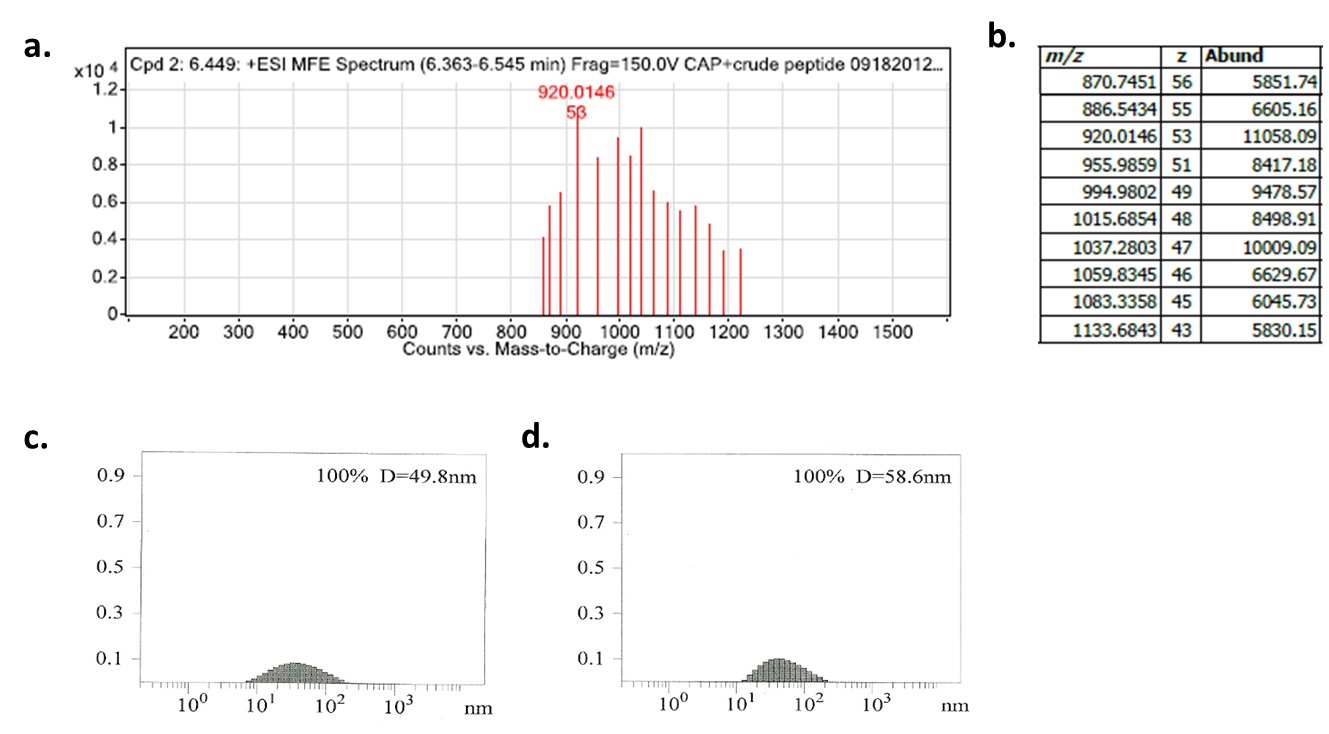
**

**Figure 4.**


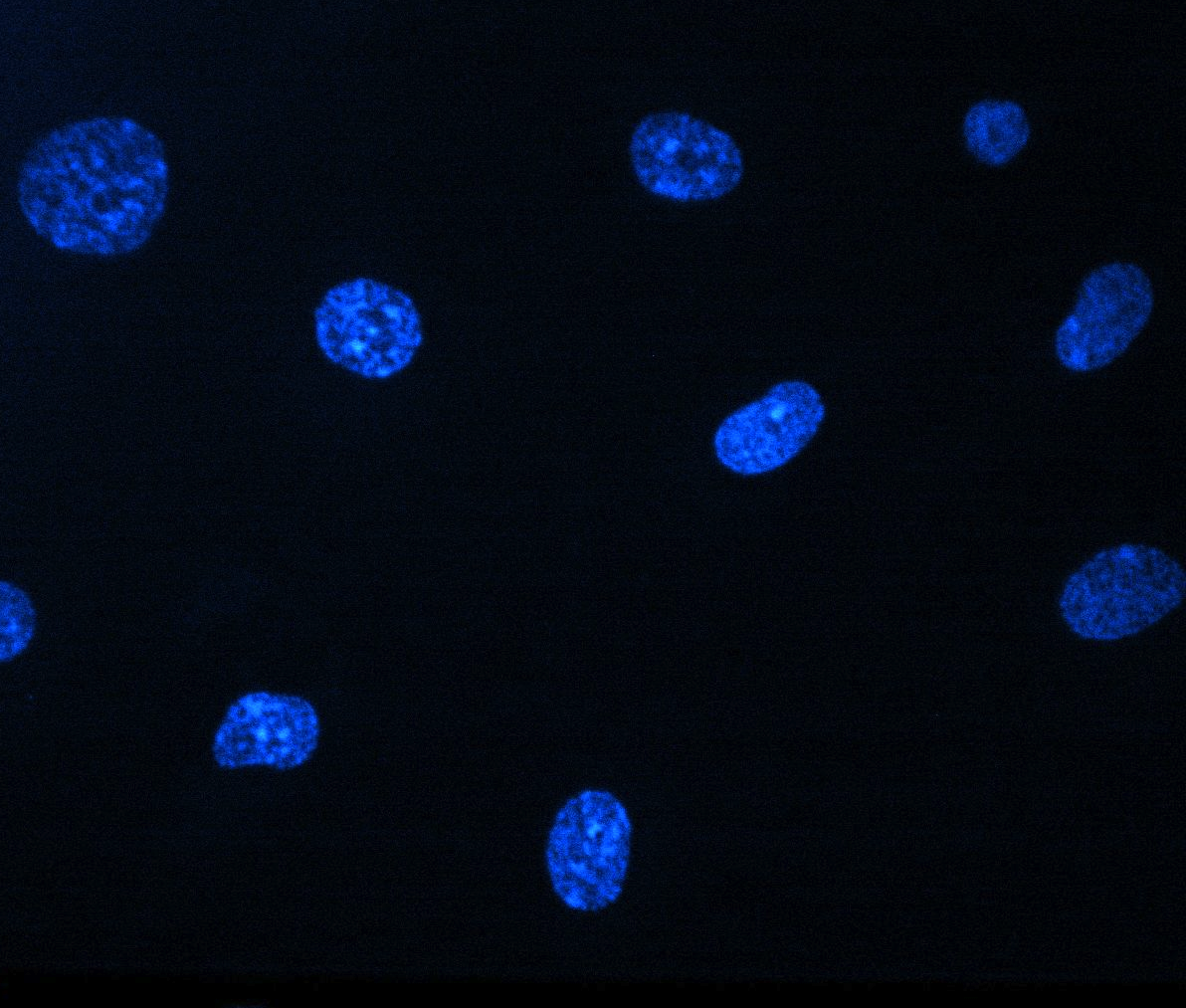

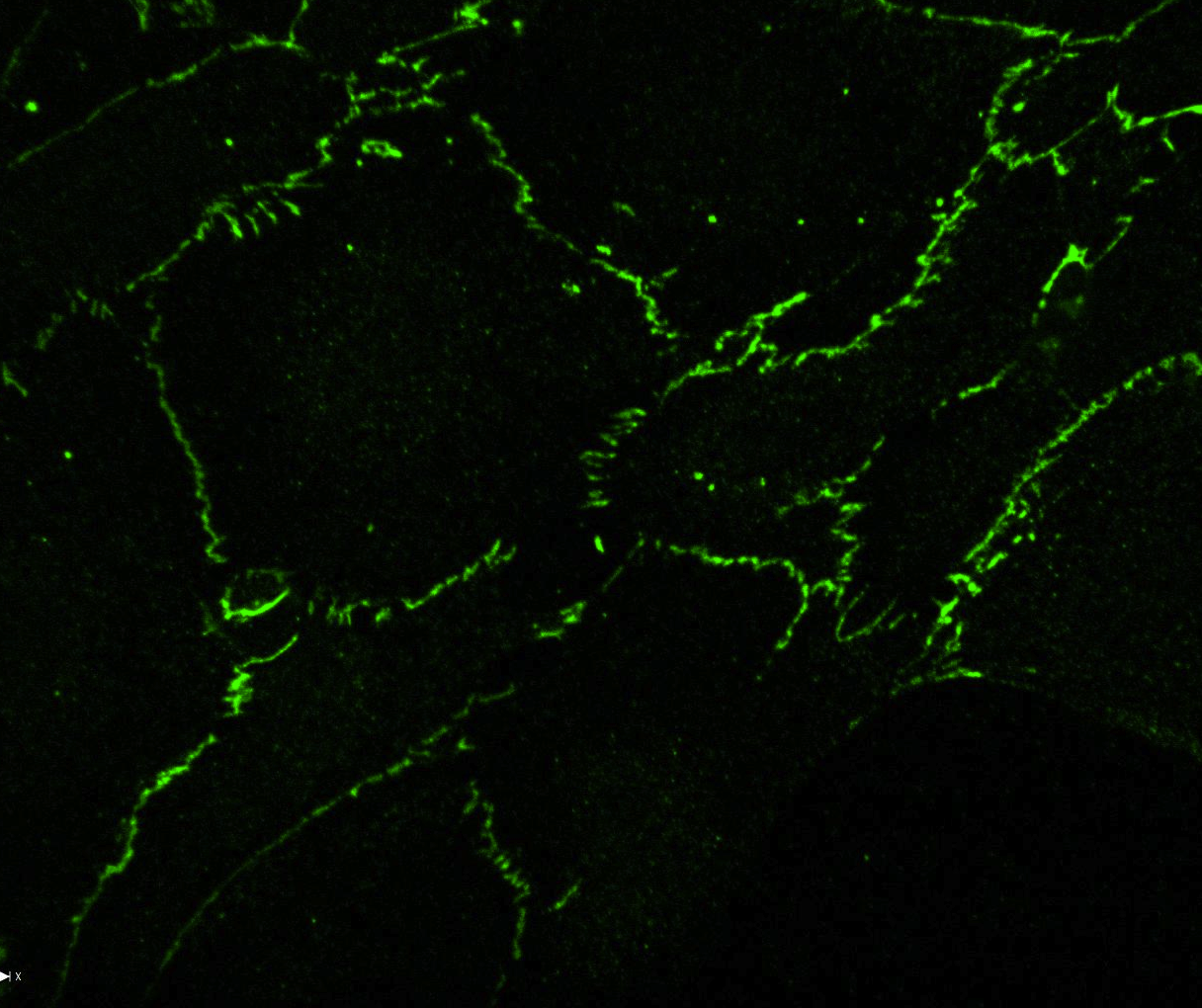

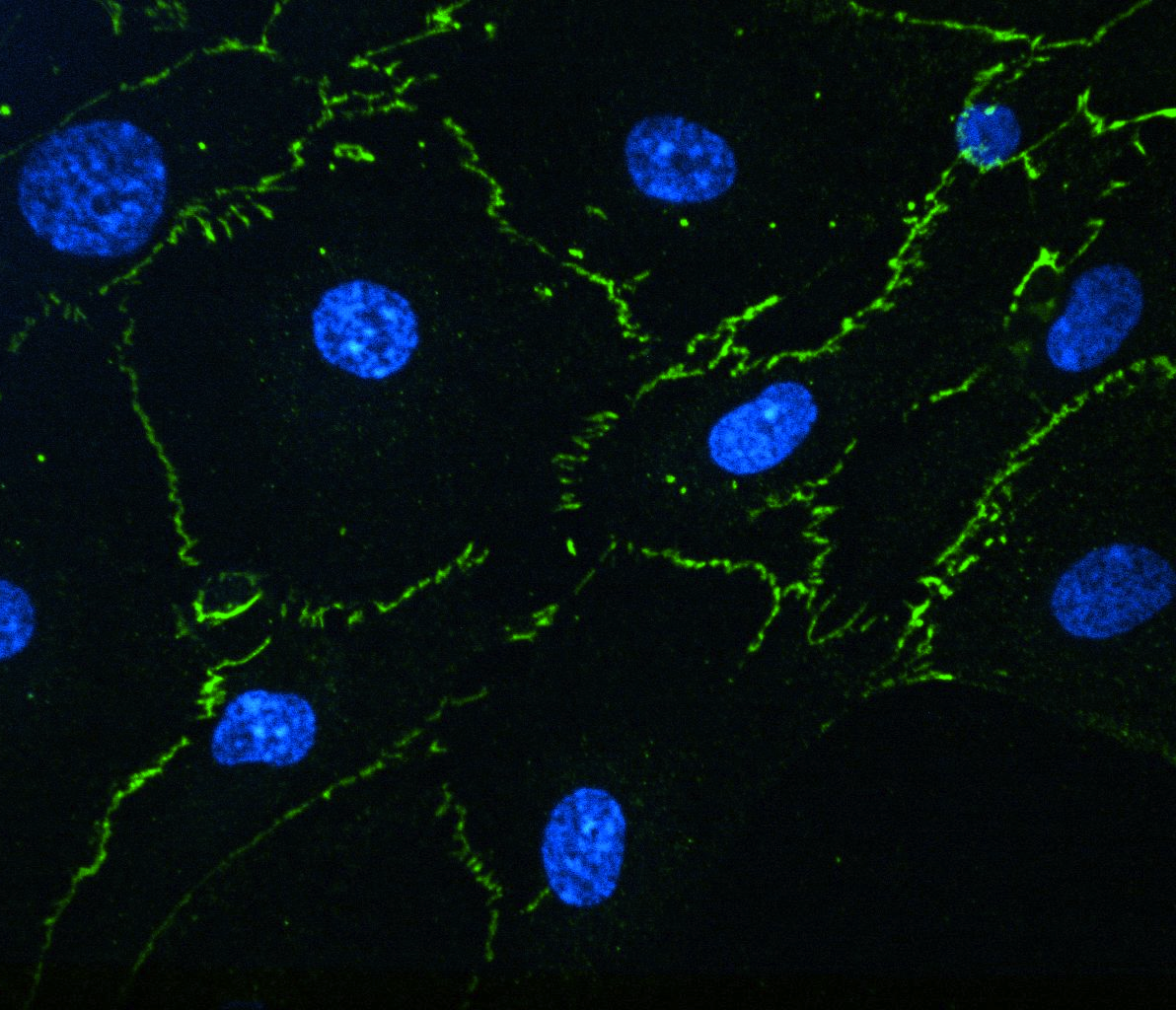


**c**

**b**

**a**

MF3 CF7 MF7


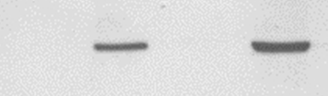

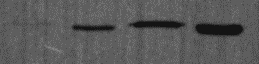


**d.**

P-gp

β-Actin

**Figure 5.**


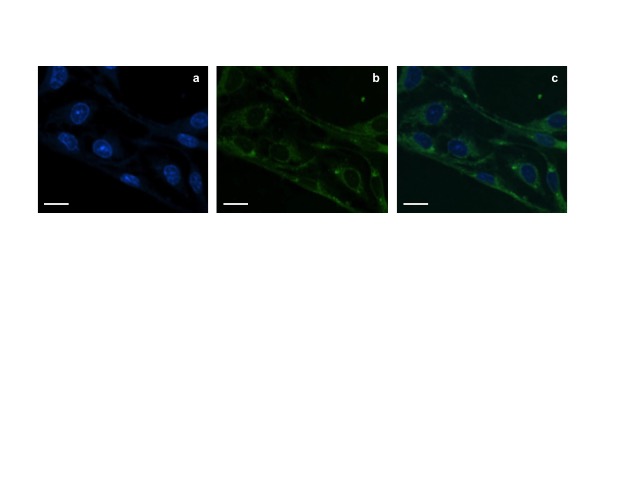


**Figure 6.**

**
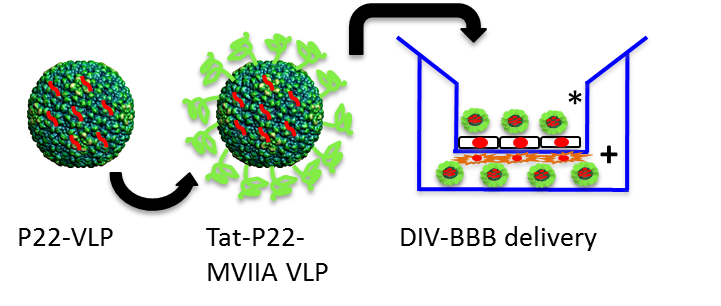
**

**a.**

**
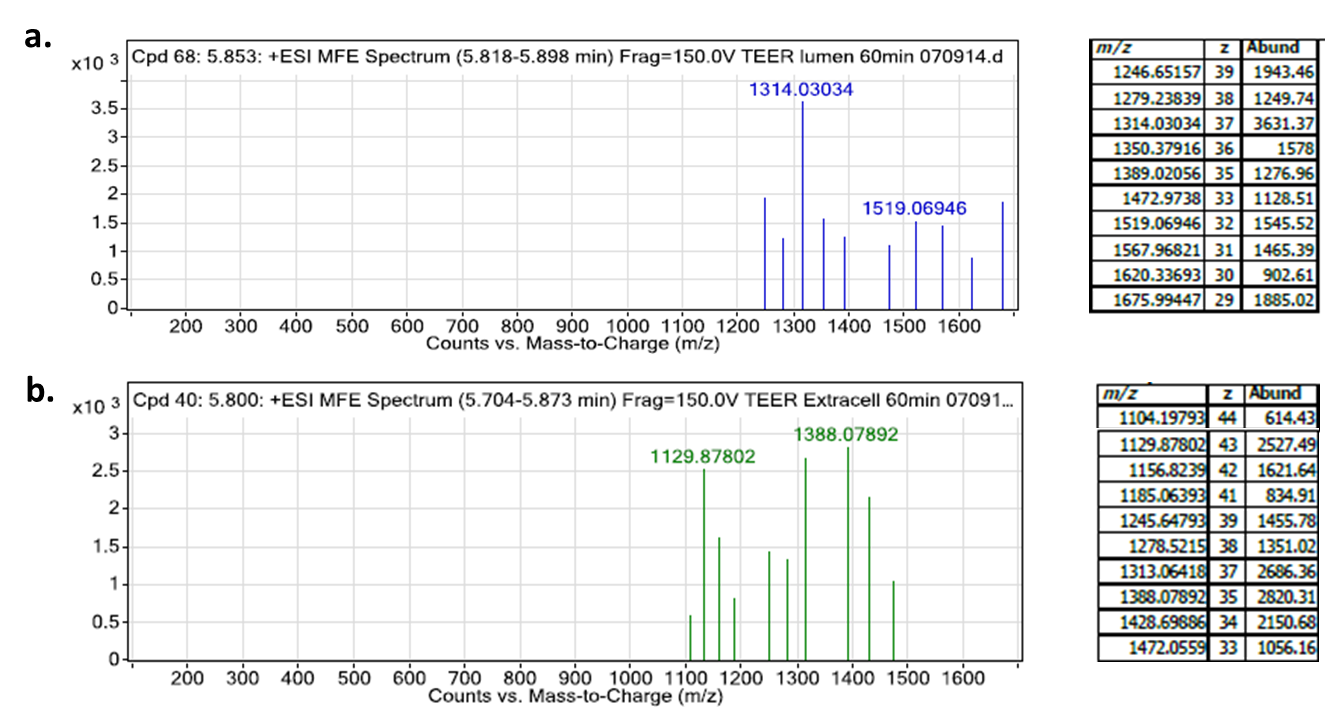
**

**b.**


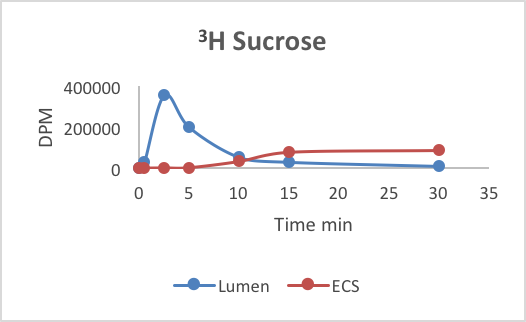


**c.**

**Figure 7.**


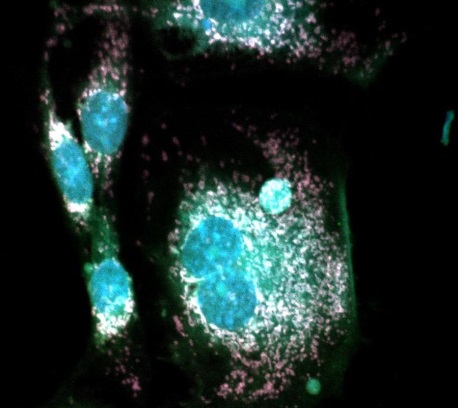

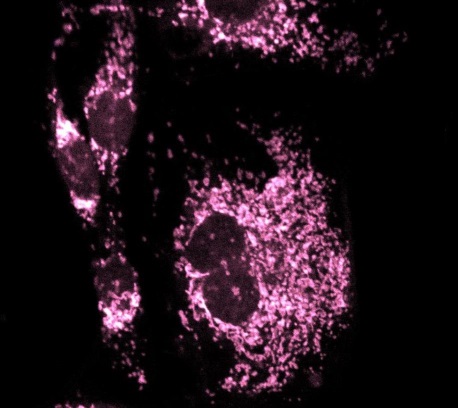

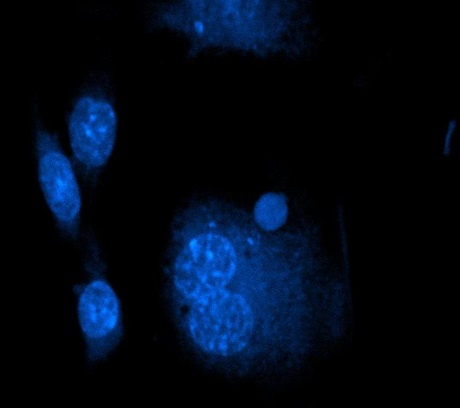

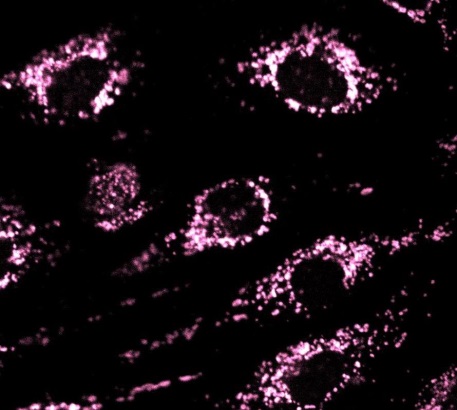

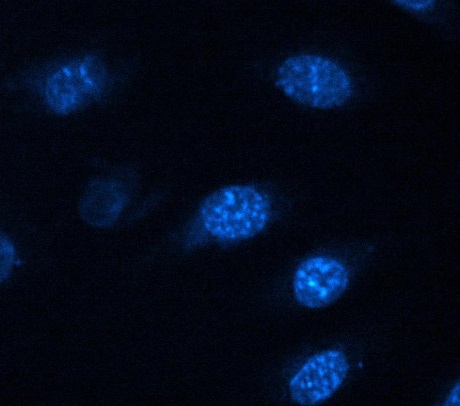

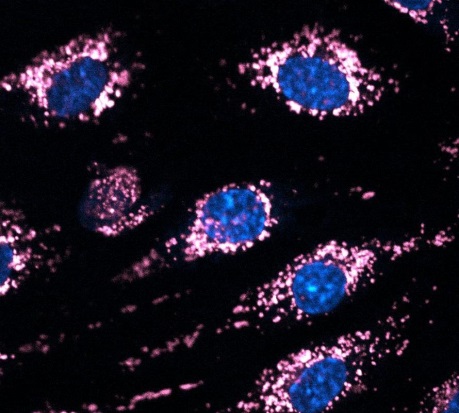


**a**

**e**

**d**

**c**

**b**

**f**

**g.**

**
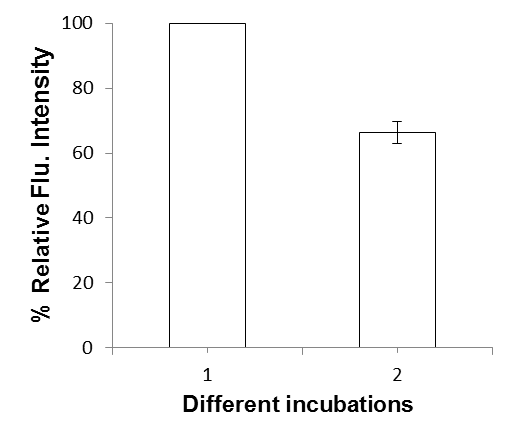
**

**Figure 8.**

**
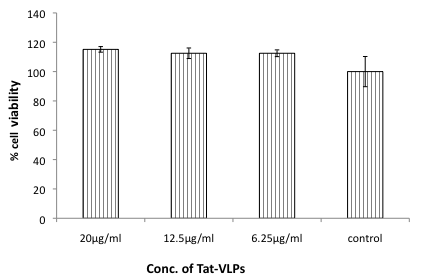
**

**Figure 9.**


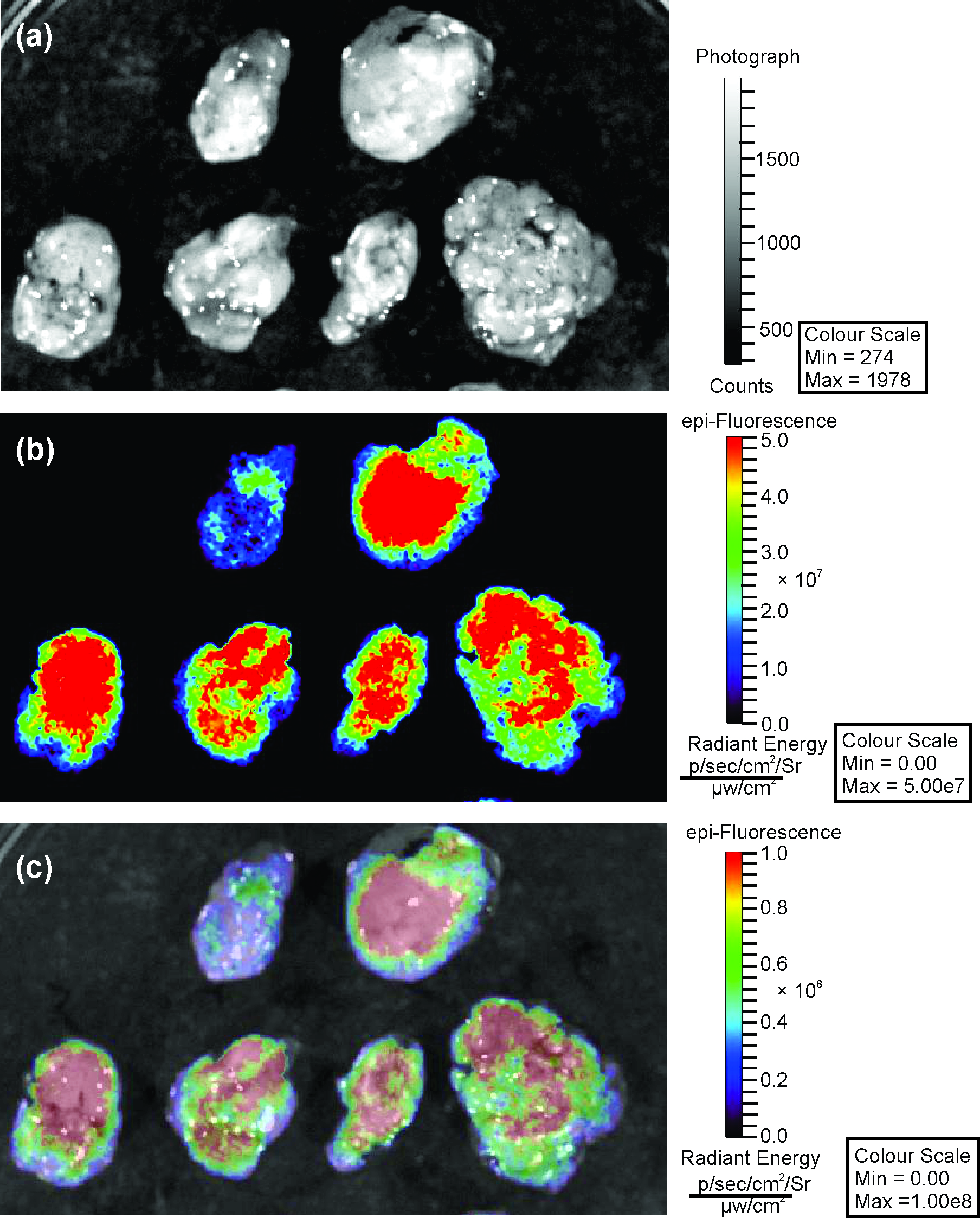


**Table 1.**

| **Bolus** | **MASSES FOUND (b/w 48-48.9kD)** | |
| --- | --- | --- |
|  | 48.37,48.85,48.97 | |
| **Time (min)** | **Luminal side** | **Extraluminal side** |
| zero | 48.1, 48.6, 48.8 | - |
| 10 | 48.6 | - |
| 15 | 48.6 | 48.5 |
| 30 | 48.4, 48.8, 48.9 | 48.4, 48.8, 48.9 |
| 60 | 48.6, 48.1, 48.8 | 48.6, 48.8 |
| 120 | 48.8 | 48.4 |
| 180 | 48.9 | 48.6, 48.0 |
